# 86 Hypothyroidism Associated with Increased Risk of Developing Contractures and Skin Disorders in Severe Burn Patients

**DOI:** 10.1093/jbcr/irae036.085

**Published:** 2024-04-17

**Authors:** Isabel B Obias, Dalton Amador, Carolina Segura, Yash Ramgopal, Chris K Soudah, Juquan Song, Amina E I ayadi, Georgiy Golovko, Steven E Wolf

**Affiliations:** University of Texas Medical Branch, Houston, TX; University of Texas Medical Branch, Galveston, TX; The University of Texas Medical Branch at Galveston, Galveston, Texas; University of Texas Medical Branch, Blocker Burn Unit, Galveston, TX; University of Texas Medical Branch, Houston, TX; University of Texas Medical Branch, Galveston, TX; The University of Texas Medical Branch at Galveston, Galveston, Texas; University of Texas Medical Branch, Blocker Burn Unit, Galveston, TX; University of Texas Medical Branch, Houston, TX; University of Texas Medical Branch, Galveston, TX; The University of Texas Medical Branch at Galveston, Galveston, Texas; University of Texas Medical Branch, Blocker Burn Unit, Galveston, TX; University of Texas Medical Branch, Houston, TX; University of Texas Medical Branch, Galveston, TX; The University of Texas Medical Branch at Galveston, Galveston, Texas; University of Texas Medical Branch, Blocker Burn Unit, Galveston, TX; University of Texas Medical Branch, Houston, TX; University of Texas Medical Branch, Galveston, TX; The University of Texas Medical Branch at Galveston, Galveston, Texas; University of Texas Medical Branch, Blocker Burn Unit, Galveston, TX; University of Texas Medical Branch, Houston, TX; University of Texas Medical Branch, Galveston, TX; The University of Texas Medical Branch at Galveston, Galveston, Texas; University of Texas Medical Branch, Blocker Burn Unit, Galveston, TX; University of Texas Medical Branch, Houston, TX; University of Texas Medical Branch, Galveston, TX; The University of Texas Medical Branch at Galveston, Galveston, Texas; University of Texas Medical Branch, Blocker Burn Unit, Galveston, TX; University of Texas Medical Branch, Houston, TX; University of Texas Medical Branch, Galveston, TX; The University of Texas Medical Branch at Galveston, Galveston, Texas; University of Texas Medical Branch, Blocker Burn Unit, Galveston, TX; University of Texas Medical Branch, Houston, TX; University of Texas Medical Branch, Galveston, TX; The University of Texas Medical Branch at Galveston, Galveston, Texas; University of Texas Medical Branch, Blocker Burn Unit, Galveston, TX

## Abstract

**Introduction:**

Patients who suffer severe burns are prone to develop various musculoskeletal and dermatological complications in the years following their injury. Hypothyroidism is classically associated with a number of muscle and skin disorders. The aim of this study is to explore the association between hypothyroidism and the emergence of these complications in severe burn patients.

**Methods:**

Severe burn patients, defined as those with burns encompassing at least 20% total body surface area (TBSA), were identified in a research database that provides access to de-identified medical records. These patients were categorized into two groups based on whether they had previously been diagnosed with hypothyroidism. Risk ratios and risk differences of contractures, including those of the muscle, joint, and skin; atrophic disorders of the skin, including hypertrophic scarring and keloid formation; and rhabdomyolysis occurring within three years of insult between the two cohorts were generated using the database’s measure of association analysis. One-year mortality between the two groups was also analyzed.

**Results:**

A total of 10,662 severe burn patients across 59 healthcare organizations were identified. The cohort with a previous diagnosis of hypothyroidism (N = 325) and the cohort without a previous diagnosis of hypothyroidism (N = 10,194) were balanced using (1:1) propensity score matching for age, sex, race, ethnicity, and TBSA.

The hypothyroidism cohort showed a greater risk of developing contractures (risk ratio [RR], 1.545; 95% confidence interval [CI], [1.094, 2.184]; p = 0.012) and atrophic skin disorders (RR, 1.502; CI, [1.002, 2.252]; p = 0.047) within three years of injury. There was no statistically significant difference in rhabdomyolysis between the two groups (RR, 1.000; CI, [0.422, 2.369]; p = 1). The hypothyroidism cohort showed a decreased risk of one-year mortality (RR, 0.589; CI, [.441, .789]; p < 0.001).

**Conclusions:**

Hypothyroidism in severe burn patients is associated with an increased risk of developing contractures and atrophic disorders of the skin in the three years after their injury. It was not associated with an increased risk of rhabdomyolysis during this same timeframe but was associated with a decreased risk of mortality within one year of injury.

**Applicability of Research to Practice:**

Hypothyroidism in severe burn patients may be regarded as a risk factor for musculoskeletal and dermatological complications pending further research. The association between hypothyroidism and lower mortality in this same population may be explored and guide the discovery of new treatments.

